# Vector-flow imaging of slowly moving *ex vivo* blood with photoacoustics and pulse-echo ultrasound

**DOI:** 10.1016/j.pacs.2024.100602

**Published:** 2024-03-15

**Authors:** Caitlin Smith, Jami Shepherd, Guillaume Renaud, Kasper van Wijk

**Affiliations:** a Department of Physics, University of Auckland, Private Bag 92019, Auckland, 1010, New Zealand; b The Dodd-Walls Centre for Photonic and Quantum Technologies, Auckland, New Zealand; c Department of Imaging Physics, Delft University of Technology, Delft, 2628 CN, The Netherlands

**Keywords:** Photoacoustic vector-flow, Velocimetry, Flowmetry, Vector-flow imaging, Blood flow, Hemodynamics, Perfusion, Ultrasound vector-flow

## Abstract

We present a technique called photoacoustic vector-flow (PAVF) to quantify the speed and direction of flowing optical absorbers at each pixel from acoustic-resolution PA images. By varying the receiving angle at each pixel in post-processing, we obtain multiple estimates of the phase difference between consecutive frames. These are used to solve the overdetermined photoacoustic Doppler equation with a least-squares approach to estimate a velocity vector at each pixel. This technique is tested in bench-top experiments and compared to simultaneous pulse-echo ultrasound vector-flow (USVF) on whole rat blood at speeds on the order of 1 mm/s. Unlike USVF, PAVF can detect flow without stationary clutter filtering in this experiment, although the velocity estimates are highly underestimated. When applying spatio-temporal singular value decomposition clutter filtering, the flow speed can be accurately estimated with an error of 16.8% for USVF and −8.9% for PAVF for an average flow speed of 2.5 mm/s.

## 1 Introduction

Photoacoustic (PA) imaging combines the specificity of optical absorption-based techniques with the centimetre imaging depths and sub-millimetre resolution of ultrasound (US). The optical biological window between 600 and 900 nm is particularly useful for imaging blood cells using PA imaging, as the absorption of light by both oxygenated and deoxygenated states of hemoglobin is orders of magnitude greater than the absorption by surrounding chromophores in soft tissues such as water [1].

Recently, PA imaging has demonstrated potential for blood flow analysis [2]. This capability is clinically important for the diagnosis of abnormalities and diseases including angiogenesis [3], burn healing [4], [5], and cancer malignancy [6]. US imaging is a staple modality for clinical blood flow measurements, but back-scattered signal from respiratory or cardiac motion (referred to as clutter) make flow quantification of blood circulation on the order of 1 mm/s challenging with clinical systems [7], [8], [9]. For PA blood flow measurements, the strong optical absorption by hemoglobin translates to a high ratio of signal-to-clutter. This is advantageous for applications with slow blood flow, where separating signal from clutter is challenging with US [10], [11].

Several PA flowmetry techniques have been developed for both the optical-resolution and acoustic-resolution regimes [2]. In the optical-resolution regime, a focused laser spot provides high-resolution imaging, but measurements are limited to shallow depths before optical diffusion becomes significant [2], [12]. Alternatively, the acoustic-resolution regime uses diffuse light to excite chromophores at multiple centimetre depths and is, therefore, more suitable for mapping of blood flow in deeper tissues. The PA signal is recorded either with a focused US transducer or a transducer array, and the resolution is limited by the acoustic wavelength. One example of acoustic-resolution PA flowmetry uses density tracking [12], [13], [14], [15], [16], [17]. These techniques use time-of-flight information between raw PA signals or images formed from consecutive laser pulses. The angle between the transducer and flow direction must be known *a priori*, which is challenging in a clinical setting in the presence of complex flow geometries and/or unresolved blood vessels. More recently, optical tracking techniques have been applied to acoustic-resolution PA images for pixel-wise velocity mapping. Zangabad et al. [18] presented a technique adapted from speckle decorrelation, while de Hoop et al. [19] used 2D cross-correlations to track speckle movements. Zhang et al. [20] utilised the optical Farneback technique translated from optical velocimetry to measure venous flow. Recently, we presented a phase-based vector-flow technique with blood-mimicking fluids [21], which in principle will be suitable for measurements of both arterial and venous flow.

In this article, we present a photoacoustic vector-flow (PAVF) technique using a least-squares estimation of the Doppler equation to quantify the magnitude and direction of flow at each pixel in acoustic-resolution PA images [21]. Our technique is validated using experimental data obtained from a blood vessel phantom containing *ex vivo* whole rat blood. We compare these results to multi-angle plane-wave US vector-flow (USVF) [22], [23], [24], [25], [26], [27], [28] for flow speeds on the order of 1 mm/s.

## 2 Theory

### 2.1 Least-squares approach for multi-angle photoacoustic vector-flow mapping

PA waves are generated when a short pulse of laser light is absorbed by chromophores, such as red blood cells, causing transient heating. These absorbers therefore expand, relax, and contract, resulting in the emission of broadband acoustic waves [1].

**Fig. 1:**
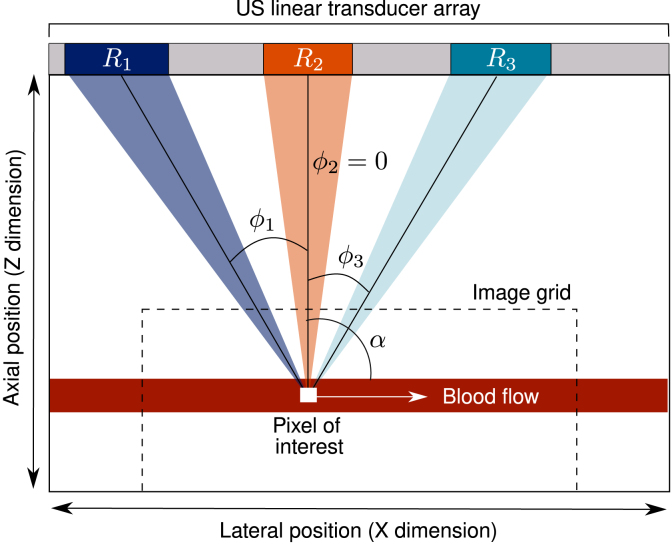
Definition of receiving angles ($\phi_{n}$) and receive apertures ($R_{x}$) used for reconstructing a single pixel. The coloured boxes labelled as “$R_{1-3}$” show subsets of transducer array elements used to observe the pixel from three different observation angles. Fewer elements are used when the pixel is nearer to the transducer in order to ensure a constant F-number in the image during reconstruction.

Consider an optical absorber moving in a homogeneous medium at a velocity v and angle α through a pixel (x, z). A local relative phase difference (ΔΨ/2π) between two frames can be converted into a displacement, U, via the spatial period (SP) in the image. This is described below in Eq. (1) and shown in Fig. 1, where ϕ is the angle between the receive direction and the transducer surface normal.

(1) $$\frac{\Delta \Psi }{2\pi }=\frac{\text{U}}{\text{SP}}\cos\left(\phi -\alpha \right).$$

The spatial period of a PA image is given by $\text{SP}=c/f_{0}$, where c is the speed of sound in the medium and $f_{0}$ is the detected centre frequency of the PA waves. Note that the spatial period of an US image is given by $c/2f_{0}$.

Eq. (1) can be rewritten using the difference of cosines trigonometric identity:

(2) $$\frac{\Delta \Psi }{2\pi }=\frac{\text{U}}{\text{SP}}\left[\cos\phi \cos\alpha +\sin\phi \sin\alpha \right].$$

Substituting in the axial (${\text{U}}_{z}=\text{U}\cos\alpha$) and lateral (${\text{U}}_{x}=\text{U}\sin\alpha$) displacement components, we can obtain the so-called “PA Doppler equation”:

(3) $$\frac{\Delta \Psi }{2\pi }=\frac{1}{\text{SP}}\left[{\text{U}}_{z}\cos\phi +{\text{U}}_{x}\sin\phi \right].$$

We can solve Eq. (3) using a least-squares approach adapted from the method first introduced by Xu et al. [29] and Maniatis et al. [30] for US imaging, and developed for USVF by Yiu et al. [27], [31]. For PAVF, we observe a pixel from several angles, $\phi_{n}$, to obtain multiple estimates of the phase change. Averaging these estimates over an ensemble of consecutive frames results in a mean phase shift $\bar{\Delta \Psi }$. Using three or more receiving angles, an over-determined system of linear equations can be generated (Eq. (4)):

(4) $$\left[\begin{array}{cc} \cos\phi_{1} & \sin\phi_{1}\\ \vdots & \vdots \\ \cos\phi_{n} & \sin\phi_{n} \end{array}\right]\left[\begin{array}{c} {\text{U}}_{z}\\ {\text{U}}_{x} \end{array}\right]=\frac{\text{SP}}{2\pi }\left[\begin{array}{c} {\bar{\Delta \Psi }}_{1}\\ \vdots \\ {\bar{\Delta \Psi }}_{n} \end{array}\right].$$

Eq. (4) is a system of linear equations of the form Ax=b, therefore the displacement components contained in $x={\left[{\text{U}}_{z}{\text{U}}_{x}\right]}^{T}$ are found by minimising ‖Ax−b‖.

When the frame rate, FR, is known, the displacement can be converted into a velocity v=U×FR. For PA imaging, the frame rate is given by the laser pulse repetition frequency.

## 3 Experimental methods

### 3.1 Experimental setup

**Fig. 2:**
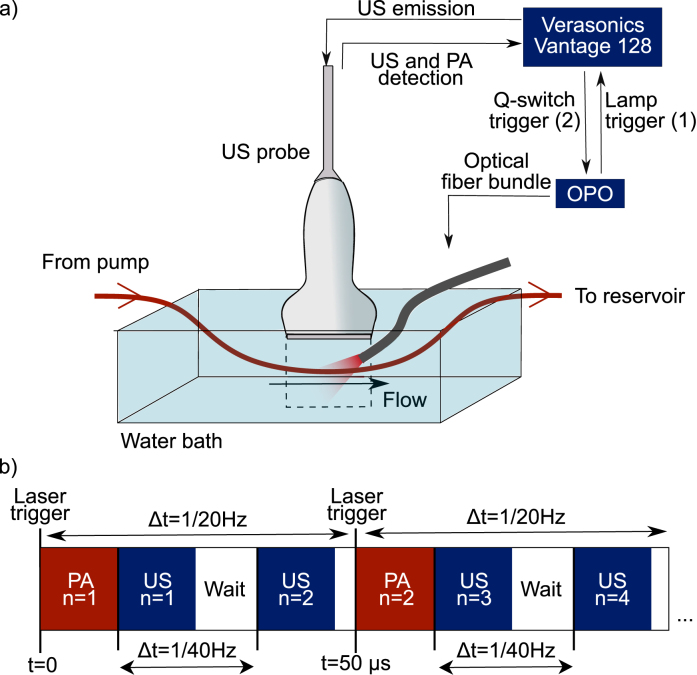
**(a)** Experimental setup for interleaved PAVF and USVF experiments. The blood is pumped between the syringe pump and the reservoir through PVC tubing. The US transducer is suspended at the surface of the water bath and the laser light is directed to the tubing via an optical fibre bundle. **(b)** Timing sequence for simultaneous acquisition of PA and US frames, indexed by “n”. Each red box represents the acquisition of a PA frame from a single laser pulse. Each blue box contains one US frame composed of five steered plane wave transmissions.

We performed PAVF and USVF measurements of whole rat blood in a blood-vessel phantom. The experimental setup is shown in Fig. 2a and the parameters used are in Table 1.

The rats were anaesthetised and euthanized for a separate project and blood was collected to reduce the number of animals used for testing in line with the policy from the University of Auckland Animal Ethics Committee. The blood was collected into heparin tubes, refrigerated, and used within 24 hours.

The blood was pumped through transparent PVC polymer tubing (inner diameter 1/16”, outer diameter 1/8”, Sigma Aldrich) in a room-temperature water bath using a syringe pump (New Era NE-4000) as shown in Fig. 1a. The pump has a dispensing accuracy of 1%, while the syringe barrel diameter has an uncertainty of 0.4%, giving an accuracy of ±1.28% for the flow speed dispensed. Before each measurement, we waited 20 s to ensure the laminar flow profile was fully developed.

The maximum flow speed, $v_{max}$, that can be measured with PAVF without aliasing is limited by the Nyquist-Shannon sampling theorem. For PAVF this is given by

(5) $${\text{U}}_{max}=\frac{\text{SP}}{2\times \cos\left(\phi -\alpha \right)}$$

$$v_{max}={\text{U}}_{max}\times \text{FR}.$$

Therefore, we used flow speeds corresponding to average volumetric flow speeds ($v_{vol.ave.}$) from 0.5 to 2.5 mm/s in increments of 0.5 mm/s. Each flow speed was repeated five times, with both the laser and US transducer realigned between trials.

### 3.2 Imaging system

For PA generation, an optical parametric oscillator (OPO, Opotek RADIANT 532 LD Tunable Optical Parametric Oscillator Laser, Carlsbad, CA) was tuned to 880 nm with a 5 ns pulse width and a repetition frequency of 20 Hz. The optimum wavelength was determined by acquiring PA images using wavelengths from 750–1000 nm. The wavelength that generated the greatest PA amplitude per unit of energy was 880 nm. The laser peak power at the fibre output was 22.1 ± 2.3 mJ/cm${}^{2}$, with a spot size of ∼5 mm from the fibre bundle.

The L11-5v transducer with a centre frequency $f_{0}=7.6$ MHz controlled by the Verasonics Vantage 128 system (Verasonics, Kirkland, WA) was used in these experiments for the transmission of the US planes waves and detection of both the PA and US signals.

We designed a custom interleaved PA and US acquisition script as shown in Fig. 2b. For every PA frame acquired, two US frames were acquired. In this way, both PAVF and USVF measured the same maximum flow speed without aliasing, as the PA spatial period is twice that of an US image. The PA FR is determined by the laser flashlamp which triggers the PA acquisition at a rate of 20 Hz [32]. The US acquisitions were separated by 25 ms to achieve an US framerate of 40 Hz. Five steered plane waves spanning 20° were transmitted for each US frame [27], with 200μs between plane wave emissions.

**Fig. 3:**
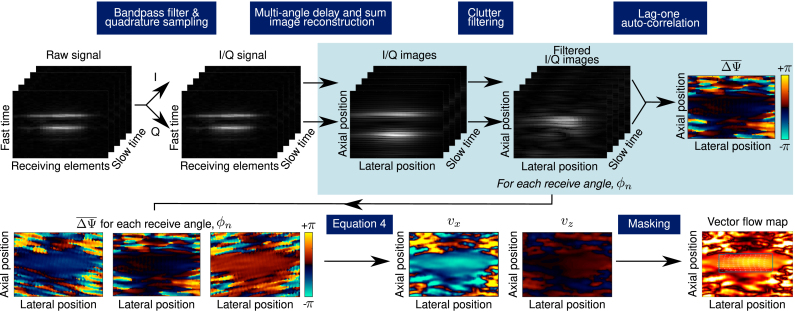
Outline of data processing steps for PA vector-flow.

## 4 Data processing

In the following section and in Fig. 3, we detail the processing pipeline beginning with the raw photoacoustic data through to generating quantitative PAVF maps.

**Table 1:** Summary of parameters used for PA and US vector-flow experiments. The fast-time bandpass filter for PAVF corresponds to the L11-5v transducer bandwidth, while the USVF filter corresponds to [0.8–1.2]$\times f_{0}$ to reduce the frequency bandwidth.

	PAVF	USVF
Number of frames	500	1000
Frame rate (FR)	20 Hz	40 Hz
Average centroid frequency	7.4 MHz	7.6 MHz
Fast-time bandpass filter	4.68−10.52 MHz	6.08−9.15 MHz
Receiving angles ($R_{x}$)	−10, −5, 0, 5, 10°	−10, −5, 0, 5, 10°
Transmission angles ($T_{x}$)	N.A.	−10, −5, 0, 5, 10°
SVD threshold	12	4
High-pass filter cut-off	$0.02\times {\text{FR}}_{PA}/2$	$0.02\times {\text{FR}}_{US}/2$

### 4.1 Generating multi-angle observations

We create multi-angle observations at each pixel by reconstructing the data with discrete receive subapertures $R_{x}$ using a modified delay-and-sum method [33], [34], [35]. The raw PA and US signals are first bandpass filtered (spectra shown in Fig. 5 with the cut-off frequencies stated in Table 1), then quadrature sampled to obtain the real (I) and imaginary (Q) components via the Hilbert transform. For each I/Q frame, an image corresponding to each $R_{x}$ is generated. The image pixel size is 50μm × 50μm.

Five $R_{x}$ ranging from $\pm 10^{\circ }$ were used for the multi-angle image reconstruction of both PAVF and USVF, resulting in 25 transmit–receive pairs for USVF, as in [27]. Increasing the total angle span improves the estimation [31], however this is typically limited to [−10° +10°] for a linear array transducer where the pitch is one wavelength. Increasing the number of subapertures improves the least-squares displacement estimates (Eq. (4)) by minimising noise which helps to ensure accurate convergence. However, this comes at the cost of increased computation time. Through empirical optimisation, we found that five subapertures offered a good balance between least-squares accuracy and computational efficiency.

The least-squares flow estimation (Eq. (4)) requires the angle between the pixel and subaperture $\phi_{n}$ to be known, and it is assumed to be constant across the entire subaperture. In practice, each subaperture observes a pixel from a range of angles, with a larger F-number (smaller subaperture) reducing the uncertainty of $\phi_{n}$ by observing the pixel with a narrower receive beam. However, increasing the F-number corresponds to a reduction in the SNR and lateral resolution. Through empirical optimisation, we found an F-number of 4 was optimal to ensure sufficient pixel SNR, which corresponds to between 13-15 elements per subaperture when the tubing is located between 15–18 mm deep. We chose to use a fixed F-number rather than a fixed subaperture size to improve the pixel SNR of pixels further away from the subaperture elements and to keep the uncertainty of $\phi_{n}$ constant throughout the image.

### 4.2 Clutter filtering

In USVF, clutter filtering is an essential step to separate background tissue from blood flow. Similarly, PA images often have strong signal generated from slow or stationary absorbers near the walls of the vessel, which need to be filtered out to extract accurate velocity estimates [12]. Clutter filtering is typically achieved by temporal high-pass filtering or spatio-temporal singular value decomposition (SVD).

#### 4.2.1 High-pass filter

In slow time (the time between consecutive images), the frequency spectrum of a pixel has a strong DC component due to constant or slowly varying pixel phase. Eq. (1) can be rewritten in terms of the slow-time (“Doppler”) frequency $f_{d}$ and flow velocity to design an appropriate cut-off threshold for the high-pass filter:

(6) $$f_{d}=\frac{\Delta \Psi }{2\pi }\times \text{FR}=\frac{\text{U}\times \text{FR}}{\text{SP}}\cos\left(\phi -\alpha \right)=\frac{v}{\text{SP}}\cos\left(\phi -\alpha \right).$$

By determining the width of the DC peak, we chose a cutoff frequency to suppress stationary signals, while retaining higher frequency content caused by flow. In this work, a 4th-order Butterworth high-pass filter was used with a cutoff frequency of 0.02×FR/2, corresponding to a cut-off frequency of 0.2 and 0.4 Hz for PAVF and USVF, respectively. This corresponds to a flow velocity of 0.04 mm/s in the axial direction.

#### 4.2.2 Singular value decomposition

Singular value decomposition (SVD) has been used for clutter filtering in US [36], [37], [38], [39] and more recently has shown to isolate spatio-temporal variability in reconstructed PA images that corresponds to the movement of absorbers [18], [40], [41].

**Fig. 4:**
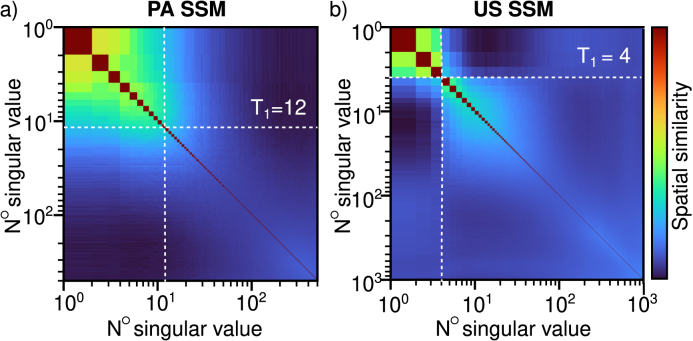
Average spatial similarity matrices (SSMs) acquired from all flow speeds and trials of the PA **(a)** and US **(b)** images used to inform the selection of the SVD thresholds. The chosen thresholds ($T_{1}$) are shown by the dashed white lines.

Using SVD, a 3D stack of reconstructed images s over slow time can be expressed as a sum of spatially separable images $I_{i}$ and temporal singular vectors $V_{i}$. $I_{i}$ is formed by reorganising the singular spatial vectors $U_{i}$ into 2D images. $I_{i}$ and $V_{i}$ are weighted by the singular values $\lambda_{i}$, which are given in descending order (i.e. the singular value with the largest magnitude is indexed “1”, while the smallest is “N”).

(7) $$s\left(x,z,t\right)=\overset{rank\left(S\right)}{\underset{i=1}{\sum }}\lambda_{i}I_{i}\left(x,z\right)V_{i}\left(t\right)$$

By removing the $I_{i}$ and $V_{i}$ that do not correspond to flow, we generate the filtered 3D image stack, $s_{filtered}$:

(8) $$s_{filtered}\left(x,z,t\right)=\overset{N}{\underset{i=T_{1}}{\sum }}\lambda_{i}I_{i}\left(x,z\right)V_{i}\left(t\right).$$

The threshold $T_{1}$ corresponds to the transition from stationary signals to flow.

In this work, we use the spatial similarity matrix (SSM) generated by computing the correlation between each $U_{i}$ [37] to inform the selection of $T_{1}$. As $I_{i}$ are ordered by decreasing magnitude of $\lambda_{i}$, the first $I_{i}$ correspond to the strong stationary signal from the edges of the tubing which have strong spatio-temporal correlations to other $I_{i}$ occupying the same space. With increasing i (decreasing $\lambda_{i}$), there is decreasing correlation between $I_{i}$ as the signals correspond to spatio-temporally uncorrelated red blood cells inside the vessel. The smallest $\lambda_{i}$ contains the most spatio-temporally uncorrelated information: noise. In general, we found the transitions between stationary, flow, and noise signals in the SSM to be more gradual for the slow-moving absorbers in our PA data (Fig. 4a) compared to the fast blood flow typically studied in clinical US [37]. For this study we used a modest $T_{1}$ to prevent over-filtering, especially as the $\lambda_{i}$ values we are filtering out are the largest, so have the strongest influence on the resulting $s_{filtered}$. The SVD cut-offs used are shown in Table 1 and the average SSMs from all experiments in Fig. 4.

### 4.3 Estimating the velocity at each pixel

The average phase difference ($\bar{\Delta \Psi }$) of a pixel over slow time is obtained using the lag-one auto-correlation method common to ultrasonic colour flow imaging. The lag-one auto-correlation [42], [43], [44], [45], [46] is given for N frames by

(9) $$\bar{\Delta \Psi }=\arctan\left[\frac{\overset{N}{\underset{i=2}{\sum }}Q_{i}I_{i-1}-Q_{i-1}I_{i}}{\overset{N}{\underset{i=2}{\sum }}I_{i}I_{i-1}+Q_{i}Q_{i-1}}\right].$$

The sign of $\bar{\Delta \Psi }$ indicates whether the movement at the pixel is towards or away from the subaperture. The multi-angle estimates of $\bar{\Delta \Psi }$ for each receiving angle $\phi_{n}$ are input into Eq. (4) and solved using the least squares approach to estimate U${}_{x}$ and U${}_{z}$ (hence, $v_{x}$ and $v_{z}$) for every pixel.

Due to the boundary buildup effect [47], the PA signal in the middle of the tube is low-amplitude (Fig. 7g) compared to the PA signal near the inner wall of the tube. In order to accurately determine the $\bar{\Delta \Psi }$ inside the tube, we must average the PA phase difference over many frames (N) to account for the lower SNR inside the tube. More frames must be used for PAVF than USVF as the SNR of blood inside the tube after clutter filtering is lower for PA than US imaging.

**Fig. 5:**
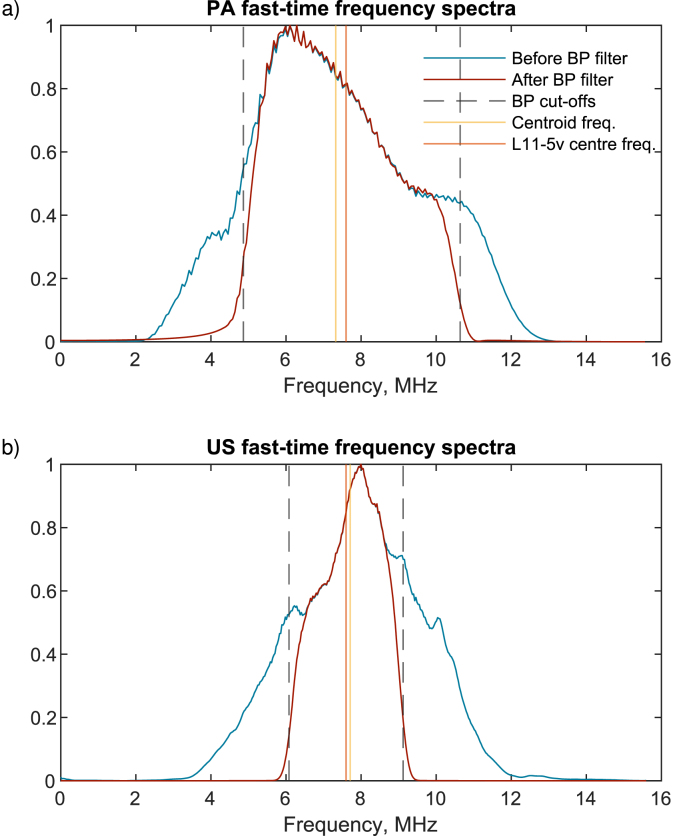
Average fast-time spectra for the first 2.5 mm/s trial for PA (top) and US (bottom) for the middle transmission angle. The dashed line shows the bandpass filter cut-offs, the orange shows the L11-5v transducer centre frequency, and the yellow line shows the centroid frequency. We can see that for the US spectrum, the transducer centre frequency and centroid frequency are much closer.

The centre frequency $f_{0}$ needed to determine the spatial period in Eq. (4) is found by determining the centroid frequency of the PA and US signals independently after band-pass filtering of the raw data (Fig. 5). Unlike US, the PA centre frequency is not well known as the PA emission is broadband in nature, so despite being convolved with transducer frequency response, the transducer centre frequency is not necessarily appropriate for PAVF. Instead, we use the detected centroid frequency ($f_{centroid}$) which is the weighted mean frequency given in Eq. (10) for a discrete spectrum of N frequencies, where $a_{i}$ is the amplitude of frequency $f_{i}$.

(10) $$f_{centroid}=\frac{\overset{N}{\underset{i=1}{\sum }}a_{i}f_{i}}{\overset{N}{\underset{i=1}{\sum }}a_{i}}$$

### 4.4 Image processing

For flow map visualisation, we masked the interior of both PAVF and USVF results by locating the inner tubing walls from the B-mode images. As the field of view is limited by the laser spot size, the length of the rectangular mask is based on the amplitude of the average image envelope of the high-pass filtered PA images for each flow speed and trial. We made a binary mask based on a threshold which was found by plotting a histogram of the envelope amplitudes and finding the envelope amplitude that corresponded to 20% of the maximum frequency on the falling edge of the histogram. Morphological transforms were used to make this binary mask smooth before the mask was confined by the tube walls found by segmentation of the US B-mode image. The ends of the mask were adjusted to make the mask rectangular before it was applied to both PAVF and USVF flow maps.

### 4.5 Flow speed calibrations

**Fig. 6:**
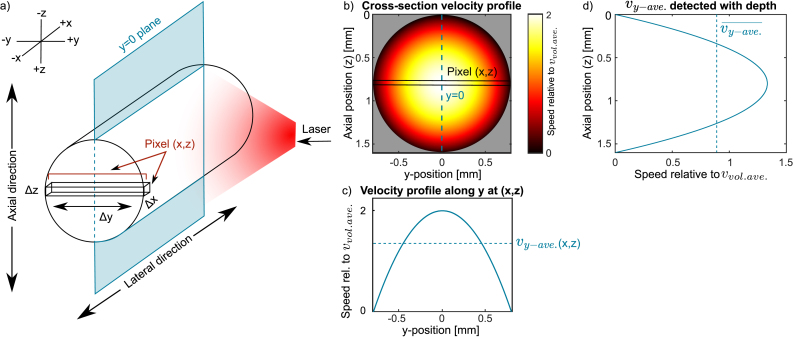
**(a)** The ultrasound transducer is aligned along the y=0 plane (shown in blue). The volume encompassed by an arbitrary pixel is highlighted by the box labelled ”Pixel(x,z)“. **(b)** Flow speed profile across the tubing cross-section. **(c)** For a pixel (x,z) at the widest point of the tube, the flow profile is shown along the y-direction. The dashed line shows the y-averaged velocity that we would expect to measure for this pixel ($v_{y-ave.}\left(x,z\right)$) **(d)** Expected flow speeds detected with depth to demonstrate what we expect to measure with 2D vector flow techniques. The dashed blue line shows the mean expected flow speed ($\bar{v_{y-ave.}}$) relative to the average volumetric flow speed ($v_{vol.ave.}$) that we would expect to measure when averaging over the inside of the tubing. From this, we know that the average flow speed inside the tubing ROI that we would expect to measure is equal to 0.89$\times v_{vol.ave.}$, which is used to estimate the expected flow speeds measured by our y-averaged vector-flow techniques.

In this experiment, the elevation resolution of the probe is larger than the tubing inner diameter. As a result, our measurements compress the 3D flow information into 2D by averaging over the elevation plane (y-direction) as shown in Fig. 6a. Therefore, both PAVF and USVF estimate the y-averaged velocity at each pixel $v_{y-ave.}\left(x,z\right)$.

Due to this effect, the average flow speeds measured inside the tube ROI $\bar{v_{y-ave.}}$ must be calibrated to the true average flow speed dispensed by the pump $v_{vol.ave.}$. The latter is determined by the volume of fluid moving per second per unit area.

We estimated the expected $v_{y-ave.}\left(x,z\right)$ (blue line in Fig. 6d) by simulating the flow profile across the tubing cross-section and averaging the flow speeds along the y-direction. The expected $\bar{v_{y-ave.}}$ across the whole tube ROI is the average of $v_{y-ave.}$ values inside the tube. From our simulations, shown in Fig. 6, this corresponds to 0.89 × $v_{vol.ave.}$. This factor of 0.89 is valid for all tubing sizes as long as they are much smaller than the US elevation plane (y-direction).

## 5 Results

We evaluated PAVF and USVF for flow mapping and quantification and investigated the influence of clutter filters on the resulting velocity estimates.

**Fig. 7:**
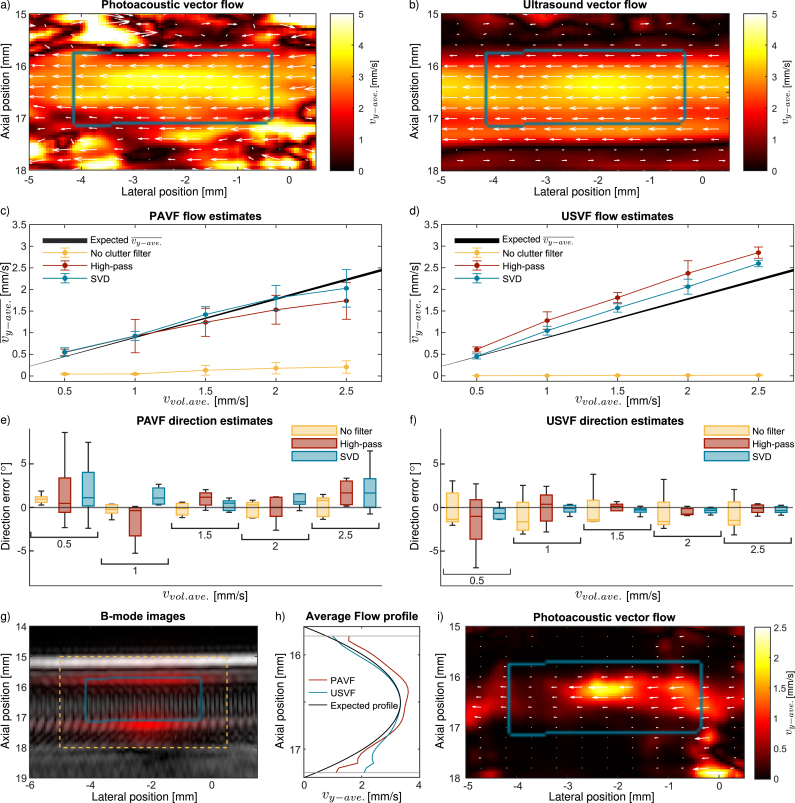
Vector-flow maps acquired using **(a)** PAVF and **(b)** USVF for a trial at an average volumetric flow speed of 2.5 mm/s with the SVD clutter filter. The colours indicate the y-averaged flow speed calculated at each pixel, while the arrows indicate the magnitude and direction of these flow speeds for points within the masked region. The blue box indicates the masked region defined by the B-mode images in **(g)** and **(i)**. Mean y-averaged flow speeds inside the tubing are shown in **(c–d)** for different pump rates and clutter filters. **(c)** and **(d)** are acquired using PAVF and USVF, respectively, for the five trials, while the error bar indicates the standard deviation. The black line shows the expected flow speed we should estimate based on the pump rate and the compression along the y-direction. The width of this line indicates the uncertainty introduced by the syringe diameter and the pump’s dispensing error. The error in the average flow direction is determined using PAVF **(e)** and USVF **(f)** for the five different flow speeds and clutter filters, indicated by the different colours. The US B-mode (grey-scale) and photoacoustic (red) B-mode images are overlaid in **(g)**. The yellow box shows the ROI for the flow maps in **(a–b, i)**, while the blue box shows the mask used to quantify the average flow speed inside the tube. Average flow speeds along the axial direction for the same 2.5 mm/s trial shown in **(a–b)** are shown in **(h)** for PAVF (red) and USVF (blue) inside the masked region. The black line indicates the expected flow profile detected with depth assuming averaging over the y-direction. The two grey lines indicate the edges of the mask. Finally, **(i)** shows the PAVF y-averaged flow map acquired without any clutter filtering for the same 2.5 mm/s trial used in **(a)**.

A parabolic flow profile can be seen in the vector-flow maps within the masked region for both the PAVF and USVF estimates (Fig. 7a and b, respectively). An area of coherent flow can be seen beneath the masked region for USVF (Fig. 7b), which is likely caused by multiple reflections at the distal wall of the tubing.

For the same trial shown in Fig. 7a–b, the average cross-sectional velocity profile detected with USVF and PAVF can be seen in Fig. 7h. Both PAVF and USVF follow the expected shape of the flow profile. However, the PAVF profile is symmetric, while USVF measures a higher velocity near the bottom wall, likely the result of the multiple reflections from the faster-moving blood at more superficial depths.

For the clutter-filtered results, the estimates acquired using USVF are faster than those acquired with PAVF for the same type of clutter filter, and these USVF velocity estimates are faster than the expected $\bar{{\text{v}}_{y-ave.}}$ values. These results are summarised in Table 2. For both PAVF and USVF, the speed estimates (Fig. 7c-d respectively) best match the expected $\bar{{\text{v}}_{y-ave.}}$ values when the SVD clutter filter is used.

**Fig. B.11:**
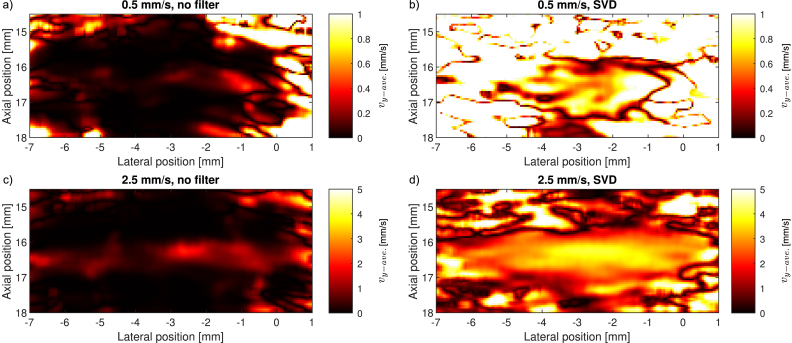
PAVF flow maps shown for two flow speeds (0.5 mm/s top, and 2.5 mm/s, bottom) of the same trial before and after SVD clutter filtering (left and right columns, respectively). The illumination was the same for these two trials.

The clutter-filtered PAVF estimates tend to be faster than the expected $\bar{v_{y-ave.}}$ values for low flow speeds and become slower relative to the expected $\bar{v_{y-ave.}}$ values with increasing $v_{vol.ave}$. This is because the clutter filters remove a greater proportion of the signal for slow flow speeds, further decreasing the already low SNR. This results in more noise in the images and causes erroneous vector-flow estimates, which increase the average flow speed inside the tubing ROI. This issue is more pronounced further away from the beam spot where the fluence and SNR is lower. Evidence for this can be seen in Appendix B by comparing two flow maps acquired for the same trial at different flow speeds, shown in Fig. B.11 before and after clutter filtering. The illumination was the same for these two trials, yet the area where coherent flow can be seen is smaller after filtering for the 0.5 mm/s trial. This implies that the clutter filtering decreases the SNR for the slower trial more than the faster trial. The same effect is not seen in USVF as the SNR is higher than PAVF. There is greater variation in flow speed estimates between trials acquired using PAVF, shown by the larger error bars in Fig. 7c compared to Fig. 7d.

The USVF direction estimates are most accurate when clutter filtering is used (Fig. 7e and f), and SVD generates more accurate direction estimates than the high-pass filter for low flow speeds. However, this difference between the two clutter filters becomes less significant with increasing flow speed, as the direction estimates improve for both filters. There is no strong trend between the accuracy of the PAVF direction estimates and flow speed (Fig. 7e). In contrast to USVF, the most accurate direction estimates are obtained when no clutter filter is used for PAVF.

Fig. 7i shows that flow is observed using PAVF without any clutter filtering, whereas we could not detect flow with USVF unless clutter filtering was utilised. This is shown for the same data set that is used to obtain the SVD-filtered results in Fig. 7(a), where $v_{vol.ave.}$=2.5 mm/s. While the flow speed estimates are much slower than expected, we can see the parabolic flow profile present within the mask. There is a fast-moving region near the centre of the image at a lateral position of −2mm, which corresponds to where the laser was incident, as shown by the red PA B-mode image in Fig. 7g. In the weakly illuminated areas, the optical penetration into the faster-flowing regions of the tubing is worse, leading to underestimation of the flow speeds for these parts of the tubing, and a weaker parabolic profile that is prone to erroneous flow vectors caused by noise.

**Fig. C.12:**
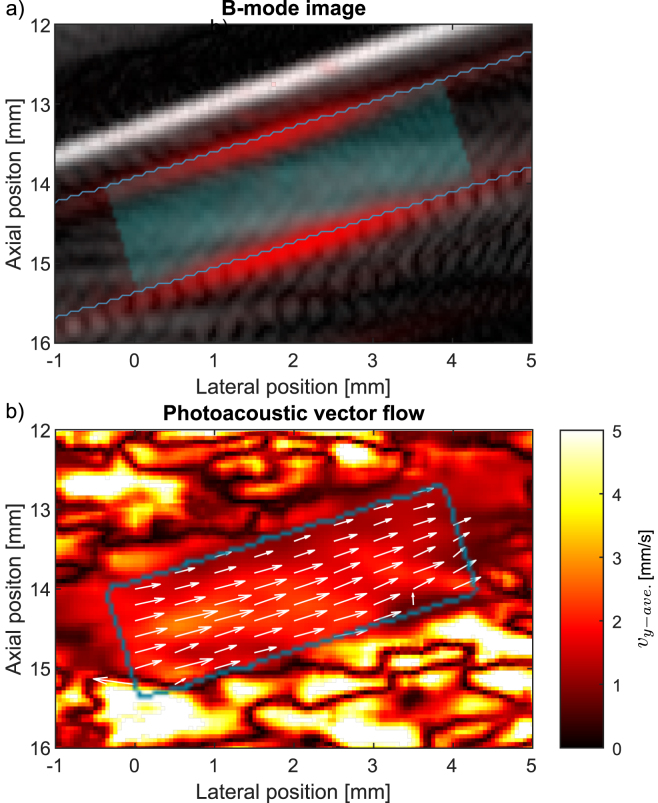
**(a)** B-mode images for an angled phantom using US (grey-scale) and PA imaging (red). The inner walls and mask as shown by the blue lines and box, respectively. **(b)** PAVF flow map for $v_{vol.ave.}$= 1.5 mm/s. The blue box is the same mask as in **(a)**.

Finally, a PAVF flow map for a phantom at 17° to the US probe is shown in Fig. C.12 to demonstrate that this technique works beyond a flat phantom.

**Table 2:**
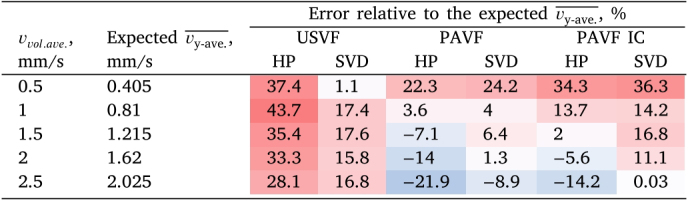
Average errors for the flow speed measurements with PAVF and USVF, and the expected $\bar{{\text{v}}_{y-ave.}}$ values, which accounts for averaging over the y-direction. These are calculated for each pump rate averaged over all five trials, both before and after illumination correction for PAVF (“IC”, described in Appendix A). The colours show errors from −50% (blue) to ＋50% (red).

## 6 Discussion

### 6.1 Velocity and direction estimates

PAVF estimates are slower than those acquired with USVF for the same type of clutter filter. We hypothesise that this is due to a bias caused by the non-uniform illumination within the tubing. When the tubing is illuminated from the side, the PA signal (which is proportional to the fluence) is predominantly generated near the tube walls, where the blood flow is the slowest due to friction with the wall. Therefore, the slow-moving flow on the walls of the tube overpowers the faster-moving flow deeper in the tube [48], resulting in the underestimation of the flow speeds [15]. In Appendix A, we have performed simulations to quantify the underestimation caused by non-uniform illumination. This process is referred to as the illumination corrected or PAVF IC results in Table 2.

It is important to note that the simulation used to determine the expected $\bar{v_{y-ave.}}$ does not consider the sensitivity of the US transducer to out-of-plane signals [49] which is relevant for modelling the expected flow speeds for USVF and PAVF. We hypothesise that the expected $\bar{v_{y-ave.}}$ values are underestimated as the simulation did not consider that the US transducer is the most sensitive to flow directly below it, so the fast flow in the middle of the tube would have a stronger influence on the $\bar{v_{y-ave.}}$ speeds detected at a pixel than the slower edges. This would explain the systematic error seen in the clutter-filtered USVF estimates where all speed estimates were faster than the expected $\bar{v_{y-ave.}}$.

Unlike US experiments, the raw PA signals generated are broadband and convolved with the transducer response upon detection. Therefore, choosing $f_{0}$ is non-trivial for PA [48]. Correctly determining this value is important as all of the displacement estimates depend on the spatial period, and therefore on the centre frequency, as seen in Eq. (1). A narrow pass-band filter of the raw RF data could better isolate the PA centre frequency, however, PA signals are lower amplitude (∼10kPa) than US (∼1MPa) [1], therefore eliminating PA signal with a filter would only further reduce the SNR. Still, compared to other PA vector-flow techniques [18], [19], [20], our experiment uses the lowest frequency transducer, which allows for better SNR at deeper depths.

For both the speed and direction estimates, PAVF has a wider range of estimates than USVF for a given pump rate and filter. This is due to the fact that for each trial, the laser and US transducer were realigned. Since the laser has a small laser spot size and a non-uniform energy density across the beam, the illumination and subsequent PA signals generated vary between trials. Conversely, we can realign the US transducer more repeatably using real-time B-mode imaging to optimise the US signal generated for each trial, leading to more repeatable results between trials.

### 6.2 Necessity of clutter filtering

Due to the background-free nature of PA imaging, PAVF can detect flow and accurately estimate flow direction without clutter filtering, unlike USVF (Fig. 7c, e, i). While USVF and PAVF both require clutter filtering to produce accurate estimates of flow velocity, the PA images inherently contain less signal clutter due to the high PA contrast of hemoglobin between 400–1000 nm [1]. This means that moving blood is the strongest source of PA waves, and therefore, PAVF estimates are less reliant on clutter filtering.

In US imaging, signal clutter refers to backscattered signal generated from sources other than blood. However, as the PA signal is only generated from blood, it raises the question, why is “clutter” filtering needed in a PA context? Because the point spread function in PA is an extended object rather than a point, the high-amplitude slowly varying boundary build-up signal leaks into the signal from deeper, low-amplitude parts of the tubing where flow is faster. To accurately determine the phase difference in the middle of the tube, clutter filtering is required to suppress this boundary build-up signal, which increases the heterogeneity of the PA images, leading to more accurate velocity estimates.

### 6.3 Choice of clutter filter

**Fig. 8:**
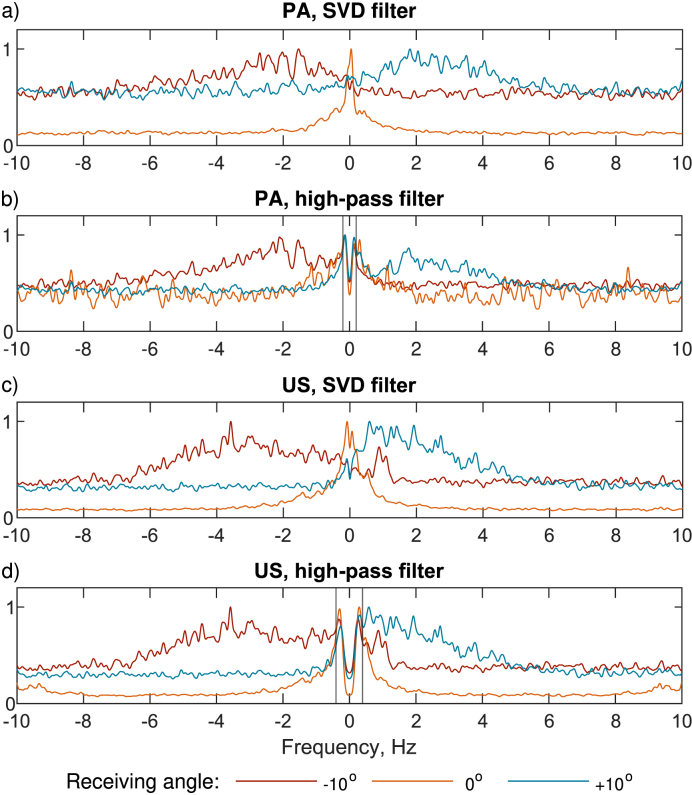
Average slow-time spectra from pixels inside the masked region shown in plots **(a–d)**, acquired after clutter-filtering for a trial at $v_{vol.ave}$=2.5 mm/s. Spectra have been smoothed to improve the visibility of peaks. **(a–b)** shows the PA slow-time spectrum detected for receiving angles $R_{x}$ = −10 (red), 0 (orange), and ＋10 degrees (blue), using the SVD filter **(a)** and high-pass filter **(b)**. **(c–d)** shows the USVF slow-time spectrum detected for the same receiving angles with a transmit angle of 0°, using the SVD filter **(c)** and high-pass filter **(d)**. The high-pass filter cut-offs are shown by the vertical lines in **(b, d)**. For both the PAVF and USVF slow-time spectra, it is possible to see the Doppler peaks due to flow at ∼−2, 0, and 2 Hz for PAVF and at ∼-4, 0, and 2 Hz for receiving angles of −10, 0 and +10°, respectively. There is a peak around 1 Hz shown for $R_{x}$=−10° US spectra **(e–f)**. This could be due to waves in the water bath moving along the tubing created during realignment of the setup for this trial.

The main difference between the slow-time spectra processed using the high-pass filter and SVD (Fig. 8), is that the SVD filter preserved the strong Doppler peaks around 0 Hz that we would expect for flow when cos(ϕ−α)≈0, ($R_{x}=0^{\circ }$), while also suppressing the DC component of the $R_{x}=\pm 10^{\circ }$ spectra. In the high-pass filtered spectra for $R_{x}=\pm 10^{\circ }$, there is still a remnant DC component that indicates that the cut-off could have been increased. However, as we are looking at low flow speeds with small Doppler frequencies, it is not possible to fully suppress the DC signal without removing the Doppler frequencies characteristic of flow. This is the main limitation of the high-pass filter: when the spectral characteristics of signal clutter (DC component) overlap those originating from the movement of blood, it is impossible to separate them temporally. We propose that for USVF, the flow estimates acquired using the high-pass filter are faster than the expected $\bar{v_{\text{y-ave.}}}$ as the low-frequency components are filtered out (Fig. 8d), so when the lag-one auto-correlator is used to determine $\bar{\Delta \Psi }$, the absence of the low-frequency components leads to $\bar{\Delta \Psi }$ being overestimated. SVD however, does not have this inherent over-estimation issue as there is no characteristic frequency cut-off associated with it, making it a promising option for low speeds and angles.

However, a concern when using SVD for quantitative flow measurements may be that the operator does not have good control over the frequency components being removed, and how this will influence the average phase differences $\bar{\Delta \Psi }$ measured. Ultimately, the choice of clutter filter and threshold can significantly bias the flow speeds estimated, so it is important for operators to understand the strengths and limitations of each filter.

For USVF, it is more straightforward to select the SVD threshold, $T_{1}$, using the SSM (Fig. 4b) than it is for PAVF (Fig. 4a). In the PA images the only signal detected is generated from the blood, so the stationary and flow spatial singular vectors both occupy similar space inside the tubing, making it challenging to identify the boundary between them. In this work, we have used the average SSM from all speeds and trials to choose a single threshold for each modality, but a more tailored approach could yield better results as different flow speeds and anatomies will generate unique SSMs. This would be aided by an automated thresholding approach, as done in US [36].

### 6.4 Towards *in vivo* translation

Several improvements are required for *in vivo* translation of this PAVF technique. The slow PRF of the laser (20 Hz) limits the flow speeds we can measure before aliasing. Therefore, faster lasers will improve the versatility of this system. Additionally, dealiasing techniques can help overcome this flow speed limit [23], [50]. Furthermore, using more frames improves the performance of the SVD clutter filter and the quality of $\bar{\Delta \Psi }$ by averaging over more frame pairs. However, the longer a scan is, the more movement there will be in the images. In these experiments, each trial of 500 frames took 25 s to acquire. Motion correction and image registration will be crucial for successful *in vivo* PAVF.

To improve the repeatability, field-of-view, and practicality for *in vivo* measurements, a fibre output that has a larger spot size and a more uniform energy distribution should be used. For example, two linear fibre optic arrays on either side of the US transducer would enable more uniform illumination across the y-direction for each pixel. To minimise PA generation from slow-moving blood on the tube walls we could use a wavelength that is more weakly absorbed by the blood to enable better transmission deeper into the tubing. There is a trade-off between imaging depth and SNR, with highly absorbing wavelengths generating strong PA signals at shallow depths and more weakly absorbing wavelengths generating lower PA amplitudes but at greater depths. Future work could determine the optimal wavelength to use *in vivo*, which minimises absorption by the skin and tissue to increase the penetration depth, but also generates sufficient PA amplitude for accurate $\bar{\Delta \Psi }$ estimates.

As described in Section 6.2, PAVF can detect flow without clutter filtering as the only signal detected by the probe is generated from blood. Future work is needed to evaluate our hypothesis that PAVF may be a better choice for detecting flow in situations where clutter filtering is difficult, such as measurements at low flow speeds with high signal clutter.

## 7 Conclusion

We have developed a PAVF technique to quantify the pixel-wise magnitude and direction of blood flow for acoustic-resolution PA imaging. We demonstrated that both PAVF and USVF can estimate flow speeds on the order of 1 mm/s for whole rat blood using a 7.6 MHz US transducer. For both modalities, the best results were obtained using an SVD clutter filter compared to a high-pass filter. While clutter filtering is essential to detect flow in USVF experiments, the background-free nature of PAVF means flow can be detected in this set-up without clutter filtering.

## CRediT authorship contribution statement

**Caitlin Smith:** Writing – review & editing, Writing – original draft, Visualization, Validation, Software, Methodology, Investigation, Formal analysis. **Jami Shepherd:** Writing – review & editing, Writing – original draft, Supervision, Software, Resources, Project administration, Methodology, Investigation, Funding acquisition, Conceptualization. **Guillaume Renaud:** Writing – review & editing, Supervision, Software, Methodology, Funding acquisition, Conceptualization. **Kasper van Wijk:** Writing – review & editing, Supervision, Resources, Methodology, Funding acquisition.

## Declaration of competing interest

The authors declare that they have no known competing financial interests or personal relationships that could have appeared to influence the work reported in this paper.

## Figures and Tables

**Fig. A.9:**
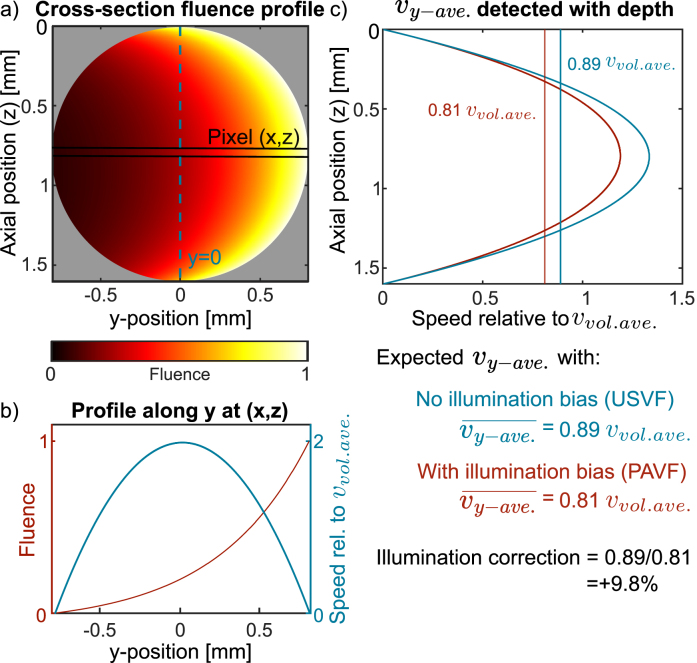
**(a)** Optical fluence profile across the tubing cross-section when illuminated from the side. **(b)** For a pixel (x,z) at the widest point of the tube, the flow profile (blue) and fluence (red) are shown along the y-direction. **(c)** Expected flow speeds detected with depth with and without considering the illumination bias (red and blue respectively), to demonstrate what we expect to measure with PAVF and USVF. The coloured vertical lines show the mean expected flow speed relative to the average volumetric flow speed that we would expect to measure when averaging over the inside of the tubing. This shows how the calibration factor of ＋9.8% is determined, which is used to quantify the underestimation of the PAVF velocity estimates caused by non-uniform illumination along the y-direction.

**Fig. A.10:**
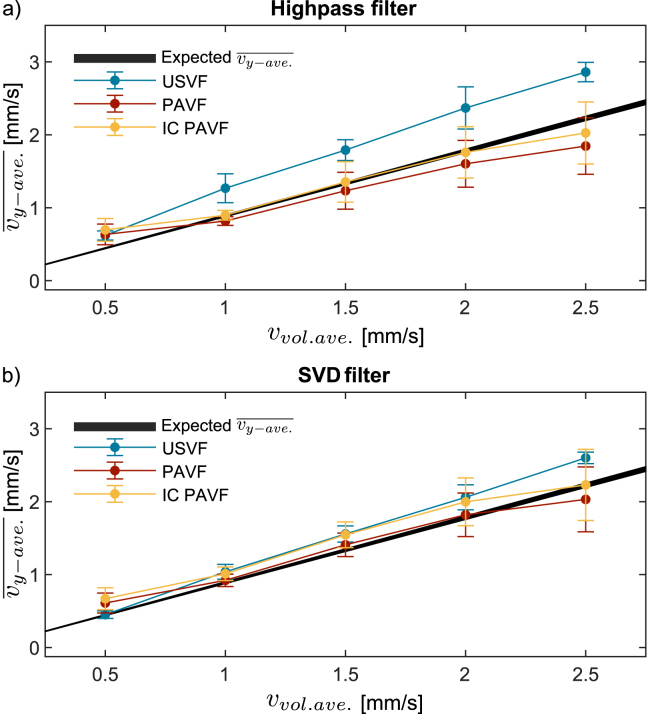
Flow speed estimates from USVF (blue), PAVF (red) and PAVF with illumination correction (yellow line, ”IC PAVF“) with the bandpass clutter filter **(a)** and SVD **(b)**. This illumination correction factor of ＋9.8% is obtained from the simulations in Fig. A.9d.

## Data Availability

Data will be made available on request.

## Appendix A Illumination correction

The pressure generated at a PA source is proportional to fluence [1]. However, when illuminating the tubing from the side, the fluence decays exponentially along the y-direction. Therefore, the velocity near the illuminated side of the tube will have a stronger contribution to the phase differnce measured than those in the centre or far side of the tube.

### A.1 Methodology

To better understand this phenomenon, we have simulated the fluence inside the tube cross-section when it is illuminated from the side (Fig. A.9a,b). An effective attenuation coefficient $\mu_{a}$ = 1.735 mm^−1^ is used, acquired from the tabulated spectral data acquired from whole human blood with SO${}_{2}>98\text{\%}$ at 880nm [51]. Then, for each depth (z-direction), the average flow speed of a pixel (x, z) is calculated by weighting the flow speeds along the y-direction by the fluence at these y-positions. From this, we simulate the flow profile at each pixel within the tube (shown by the red line in Fig. A.9c) that we expect to measure using PAVF.

From these simulations, we can account for the discrepancy between USVF and PAVF estimates due to the non-uniform fluence across the tubing cross-section. This illumination correction is given by 0.89÷0.81 (see Fig. A.9c), corresponding to an increase of 9.8% from the PAVF velocity estimates presented in Fig. 7c.

### A.2 Results

In Fig. A.10 we show the PAVF results before and after illumination correction, acquired with the high-pass filter and SVD by increasing the average flow speed estimates by 9.8%. For the fastest flow speed ($v_{vol.ave.}$=2.5 mm/s) where we would expect the PAVF estimates to be the most accurate, the estimates became closer to the expected $\bar{v_{y-ave.}}$. For lower flow speeds where more spurious vectors are present, the illumination correction increases the already overestimated flow speeds. As the illumination correction only increases the flow speed estimates by 9.8%, it is considerably less influential on the flow estimates than the clutter filter. Therefore, it is more important to carefully select an appropriate clutter filter than it is to account for this illumination bias.

The purpose of this illumination correction is to explain and attempt to quantify the differences in estimates between PAVF and USVF, rather than a fool-proof way to improve PAVF accuracy. The simulation used the optical attenuation coefficient of human blood at 880 nm rather than rat blood, which would have a different value due to a size difference (6μm compared to 7-8μm) resulting in different scattering properties. Furthermore, in *ex vivo* blood other components such as methemoglobin may be present which are not considered when calculating the effective attenuation coefficient. Finally, these simulations are overly simplified and do not consider the non-uniform beam profile, or that the middle of the laser beam is not always directly aligned with the middle of the tube in the z-direction.

## Appendix B Effect of clutter filtering on SNR

As described in Section 5, for accurate PAVF estimates, the pixel must have sufficient SNR, however, clutter filtering reduces the SNR and the area where coherent flow can be detected. This is more pronounced in slower flow speeds where the clutter and flow signal are spectrally more similar, so the clutter filter will remove a greater proportion of the signal, reducing the SNR. This is shown in Fig. B.11, since the area of coherent flow that can be seen is larger for the faster 2.5 mm/s trial than the 0.5 mm/s trial, despite the illumination and alignment being the same for these two trials.

## Appendix C Angled flow phantom

To verify that the PAVF technique can measure angled flow, we repeated the same methodology on a phantom at 17° with $v_{vol.ave.}$= 1.5 mm/s. The B-mode image and vector flow map after SVD filtering are shown in Fig. C.12.

The main consideration when acquiring data using an angled phantom is that aliasing will occur at lower flow speeds as the speed in the axial direction increases with tube angle. However, a flat phantom is more challenging as the flow will generate smaller average phase differences. This in turn makes clutter filtering more difficult because the average phase differences correspond to smaller Doppler frequencies which are more difficult to separate from signal clutter. Additionally, phased-based flow techniques are most sensitive in the axial direction, so a flat tube with flow in the lateral direction is challenging.
